# The impact of rheumatological disorders on lymphomas and myeloma: a report on risk and survival from the UK’s population-based Haematological Malignancy Research Network

**DOI:** 10.1016/j.canep.2019.02.014

**Published:** 2019-04

**Authors:** Eleanor Kane, Daniel Painter, Alexandra Smith, Simon Crouch, Steven Oliver, Russell Patmore, Eve Roman

**Affiliations:** a Department of Health Sciences, University of York, York, UK; b Hull York Medical School, York, UK; c Queens Centre for Oncology, Castle Hill Hospital, Hull, UK

**Keywords:** Chronic lymphocytic leukaemia, Diffuse large B-cell lymphoma, Follicular lymphoma, Marginal zone lymphoma, Multiple myeloma, Rheumatoid arthritis, Autoimmune disease

## Abstract

**Background:**

Autoimmune inflammatory disease increases the risk of diffuse large B-cell lymphoma (DLBCL) and marginal zone lymphoma (MZL), but findings for other mature B-cell malignancies are equivocal. Furthermore, it has been suggested that the increase in DLBCL is due to the activated B-cell (ABC) subtype; but data on this, and the impact of inflammatory co-morbidities on survival, are sparse and contradictory.

**Methods:**

Data are from an established UK population-based cohort. Patients (n = 6834) diagnosed between 01/2009 and 08/2015 are included; DLBCL (n = 1771), myeloma (n = 1760), chronic lymphocytic leukaemia (CLL, n = 1580), MZL (n = 936), and follicular lymphoma (FL, n = 787). Information on rheumatological disorders and deaths was obtained by record-linkage to nationally compiled Hospital Episode Statistics, with age-and sex-matched individuals (n = 68,340) from the same catchment population (˜4 million people) providing the comparator.

**Results:**

Significantly increased risks for DLBCL (OR = 2.3, 95% CI 1.8–2.8) and MZL (OR = 2.0, 95% CI 1.5–2.7) were found for those with rheumatological disorders; the site distribution of those with/without rheumatological conditions differing for DLBCL (p = 0.007) and MZL (p = 0.002). No increases in risk were observed for the remaining mature B-cell malignancies, and no associations with survival were detected for DLBCL (age-adjusted HR = 1.2, 95% CI 0.9–1.6) or MZL (age-adjusted HR = 1.0, 95% CI 0.6–1.9). Furthermore, whilst our findings provide evidence for an association with rheumatological disease severity for DLBCL, they offer little support for the notion that the association is driven by an increase in the incidence of the ABC subtype.

**Conclusion:**

Our findings support the hypothesis that the chronic activation and proliferation of specific B-cell populations which characterize autoimmune disease increase the potential for the lymphomagenic events that lead to DLBCL and MZL in both males and females; but have no impact on the development of CLL, FL or MM, or on survival.

## Highlights

• Diffuse-large B-cell and marginal zone lymphoma: inflammatory conditions increase risk.
• Myeloma, follicular lymphoma and chronic lymphocytic leukaemia: inflammatory conditions do not impact on risk.
• Inflammatory conditions are far more common in women, but gender is not an effect modifier.
• Inflammatory co-morbidities do not impact adversely on mature B-cell malignancy survival.
• The increased risk for diffuse large B-cell lymphoma is not mediated by the activated B-cell subtype.

## 1 Introduction

Arising from the malignant transformation of lymphoid cells, lymphoid neoplasms are the fourth most common cancer subtype in men (after prostate, lung, and colorectal) and women (after breast, lung, and colorectal) [1,2]. Including chronic lymphocytic leukaemia (CLL), the lymphomas and multiple myeloma (MM), this diverse cancer group is dominated by mature B-cell tumours, which account for around 90% of the total [[2], [3], [4]]. Immune dysregulation plays a pivotal role in the development of mature B-cell malignancies and several autoimmune conditions have been linked with increased risk. Among the strongest and most consistently reported associations are those between B-cell lymphomas – particularly diffuse large B-cell lymphoma (DLBCL) and marginal zone lymphoma (MZL) – and chronic B-cell-activating inflammatory diseases notably rheumatoid arthritis, systemic lupus erythematosus and Sjögren’s syndrome [[4], [5], [6], [7], [8], [9], [10], [11]]. Furthermore, there is some evidence to suggest that DLBCL patients with chronic inflammatory conditions are more likely to be diagnosed with the activated B-cell (ABC) subtype [5,12], which has a poorer prognosis than the germinal centre B-cell (GCB) subtype and tends to be diagnosed at a slightly older age [13,14].

More recently, interest in the impact that autoimmune conditions (and their treatments) could have on the survival of patients with mature B-cell neoplasms has been growing [[15], [16], [17], [18]]. The complexity of both groups of disorders, coupled with the rapidly changing treatment landscape, means that data on this topic are comparatively sparse, and the few studies that have investigated this issue have produced contradictory results [[15], [16], [17], [18]]. Accordingly, examining both aetiological associations and survival in a well-established contemporary population-based UK cohort of patients with haematological malignancies [19,20], the present report describes the relationship between rheumatological disorders and the five commonest mature B-cell malignancies; namely DLBCL, MZL, FL, CLL and MM.

## 2 Materials and methods

### 2.1 Setting

The study is set within the UK’s Haematological Malignancy Research Network (HMRN) which, initiated 09/2004 with a catchment population approaching 4 million accrues ˜2400 haematological malignancy diagnoses each year, provides contemporary real-world data that can be generalized to the UK as a whole (www.hmrn.org) [19]. HMRN has two population-based cohorts at its core, a patient cohort and a general population cohort; full details of the former’s structure, data collection methods, and ethical approvals are provided elsewhere [19,20]. Briefly, HMRN operates under a legal basis that permits full treatment and outcome data to be collected from clinical records without explicit consent, and both cohorts are linked to nationwide information on deaths, cancer registrations and Hospital Episode Statistics (HES) [20]. All patient cohort diagnoses, including progressions and transformations, are reported and coded to the latest WHO ICD-O3 [4] by haematopathologists at the Haematological Malignancy Diagnostic Service (HMDS); a fully integrated specialist laboratory housing all of the technology and expertise required for the diagnosis and monitoring of haematological cancers (www.hmds.info) [[21], [22], [23]]. Patient cohort members diagnosed between 01/2009 and 12/2015 were matched at the point of diagnosis on age and sex to 10 randomly selected individuals from the same catchment population from the national population-based NHS Central Register by NHS Digital (https://digital.nhs.uk/). These general population-cohort members were assigned a “pseudo-diagnosis” date corresponding with their matched cases’ diagnosis date, and none had a record of a previous cancer registration for a haematological malignancy.

### 2.2 Study population

This report includes 6834 patients newly diagnosed 01/2009-07/2015 with one of the five commonest mature B-cell malignancies, DLBCL (n = 1771), MM (n = 1760), CLL (n = 1580), MZL (n = 936), or FL (n = 787), and their age- and sex-matched counterparts from the general population cohort (n = 68,340). In addition to examining core diagnostic/prognostic clinical data, for DLBCL patients with available data/material we examined cell-of-origin assigned on the basis of: 1) immunohistochemical expression of CD10, BCL6 and IRF4/MUM1 [24]; and 2) gene-expression profiling of pre-treatment biopsies analysed using the Illumina WG-DASL and DLBCL automatic classifier (DAC) [13,25,26].

As with many other chronic diseases, hospital admissions for rheumatological disorders are comparatively rare. However, as detailed by the UK’s National Institute for Health and Clinical Care Excellence (NICE), the diagnosis and treatment of autoimmune/inflammatory conditions like rheumatoid arthritis and Sjögren’s Syndrome require specialist clinical input, and patients are diagnosed and managed as outpatients in secondary care [27,28]. Hence, for the purposes of the present analysis, data on all secondary care episodes in both inpatient and outpatient settings between April 2003 and the date of B-cell malignancy diagnosis (patient cohort members), or the corresponding pseudo-diagnosis date (comparator cohort members), were obtained for all individuals *via* linkage to HES. Within HES, each episode of hospital care is assigned to an individual consultant, which in turn is linked to their clinical specialty categorization. For the purposes of the present analysis, each rheumatology episode was defined either as a face-to-face outpatient attendance or as a hospital admission under the care of the rheumatology specialty.

A case-control approach was used to quantify associations between rheumatology episodes and malignancy; odds ratios (ORs) and 95% confidence intervals (CIs) being estimated using conditional logistic regression. Monte-Carlo simulation techniques were used to estimate p-values when cell numbers were ≤ 5 [29]. The potential impact of rheumatological disease on survival was also examined among the cases; overall survival (OS) and hazard ratios (HRs) from Cox proportional hazards models being reported. Analyses were conducted using Stata 15.1 (StataCorp 2017) and R (R Core Team 2013).

## 3 Results

**Fig. 1:**
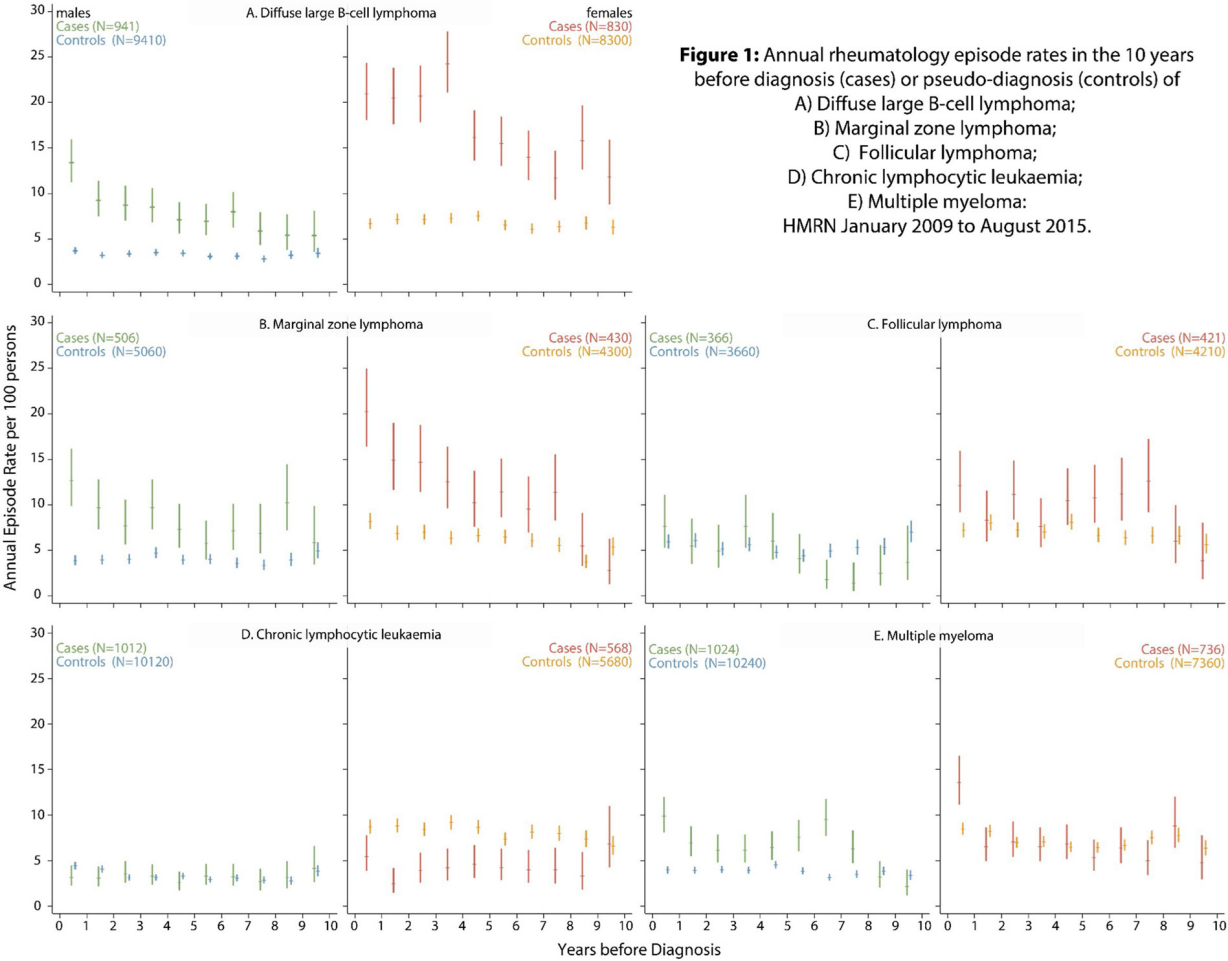
Annual rheumatology episode rates in the 10 years before diagnosis (cases) or pseudo-diagnosis (controls) of A) Diffuse large B-cell lymphoma; A) Marginal zone lymphoma; C) Follicular lymphoma; D) Chronic lymphocytic leukaemia; E) Multiple myeloma: HMRN January 2009 to August 2015.

Information on hospital episodes under the care of rheumatology before the diagnosis of a mature B-cell malignancy (cases) or corresponding pseudo-diagnosis (controls) is presented in Fig. 1 and Table 1. Fig. 1(A to E) shows the annual rheumatology episode rate of cases and controls for males and females separately; and Table 1 distributes cases and controls (both sexes combined) according to the number of episodes experienced, the top section including data on all episodes and the second and third sections excluding those that occurred within six, and then twelve, months before diagnosis/pseudo-diagnosis.

**Table 1:** Number of mature B-cell maligancy cases and controls, Odds Ratios (OR) and 95% Confidence Intervals (CI) distributed by number and timing of rheumatology hospital episodes: HMRN diagnoses 2009 to 2015.

	Diffuse large B-cell lymphoma			Marginal zone lymphoma			Follicular lymphoma			Chronic lymphocytic leukaemia			Multiple Myeloma		
	Controls	Cases	OR (95%CI)^1^	Controls	Cases	OR (95%CI)^1^	Controls	Cases	OR (95%CI)^1^	Controls	Cases	OR (95%CI)^1^	Controls	Cases	OR (95%CI)^1^
	%	%		%	%		%	%		%	%		%	%	
Total	N = 17710	N = 1771		N = 9360	N = 936		N = 7870	N = 787		N = 15800	N = 1580		N = 17600	N = 1760	
*All episodes up to diagnosis*															
None	93.3	88.1	1(ref)	93.4	86.9	1(ref)	92.9	90.5	1(ref)	93.3	93.6	1(ref)	93.2	90.3	1(ref)
≥1	6.7	11.9	1.9(1.6–2.2)	6.6	13.1	2.2(1.8–2.7)	7.1	9.5	1.4(1.1–1.8)	6.7	6.4	1.0(0.8–1.2)	6.8	9.7	1.5(1.2–1.8)
1	2.4	3.3	1.5(1.1–2.0)	2.4	4.4	2.0(1.4–2.8)	2.1	2.9	1.5(0.9–2.3)	2.3	2.5	1.1(0.8–1.5)	2.3	3.6	1.7(1.3–2.2)
2	1.1	1.3	1.3(0.8–2.0)	1.1	2.4	2.4(1.5–3.9)	1.1	1.5	1.5(0.8–2.7)	1.1	1.1	1.0(0.6–1.6)	1.0	2.1	2.2(1.5–3.2)
≥3	3.2	7.2	2.4(2.0–3.0)	3.2	6.4	2.2(1.6–2.9)	4.0	5.1	1.3(0.9–1.9)	3.3	2.8	0.8(0.6–1.2)	3.6	3.9	1.1(0.9–1.5)

*Excluding episodes within 6 months of diagnosis*															
None	93.6	89.8	1(ref)	93.7	89.1	1(ref)	93.2	91.6	1(ref)	93.6	94.1	1(ref)	93.6	93.2	1(ref)
≥1	6.4	10.2	1.7(1.4–2.0)	6.3	10.9	1.8(1.5–2.3)	6.8	8.4	1.3(1.0–1.6)	6.4	5.9	0.9(0.7–1.1)	6.4	6.8	1.1(0.9–1.3)
1	2.2	2.3	1.1(0.8–1.5)	2.2	3.4	1.6(1.1–2.4)	2.1	2.2	1.1(0.6–1.7)	2.2	2.3	1.0(0.7–1.5)	2.1	2.3	1.1(0.8–1.5)
2	1.1	1.2	1.2(0.8–1.9)	1.0	1.5	1.6(0.9–2.8)	1.1	1.4	1.3(0.7–2.4)	1.1	0.9	0.8(0.5–1.4)	0.9	1.3	1.4(0.9–2.1)
≥3	3.1	6.7	2.3(1.9–2.8)	3.1	6.0	2.1(1.5–2.8)	3.6	4.8	1.4(1.0–1.9)	3.1	2.7	0.9(0.6–1.2)	3.4	3.3	1.0(0.7–1.3)

*Excluding episodes within 12 months of diagnosis*															
None	93.9	90.2	1(ref)	93.9	89.7	1(ref)	93.5	92.2	1(ref)	93.9	94.3	1(ref)	93.8	93.6	1(ref)
≥1	6.1	9.8	1.7(1.4–2.0)	6.1	10.3	1.8(1.4–2.2)	6.5	7.8	1.2(0.9–1.6)	6.1	5.7	0.9(0.7–1.2)	6.2	6.4	1.0(0.8–1.3)
1	2.2	2.3	1.1(0.8–1.5)	2.2	3.0	1.4(1.0–2.1)	2.0	1.7	0.8(0.5–1.5)	2.1	2.2	1.0(0.7–1.4)	2.1	2.0	1.0(0.7–1.4)
2	1.0	1.2	1.3(0.8–2.0)	1.0	1.7	1.9(1.1–3.2)	1.0	1.5	1.6(0.9–2.9)	1.0	1.0	1.0(0.6–1.6)	0.9	1.4	1.5(1.0–2.3)
≥3	2.9	6.3	2.3(1.8–2.8)	2.9	5.6	2.0(1.5–2.7)	3.5	4.6	1.3(0.9–1.9)	3.0	2.5	0.8(0.6–1.2)	3.2	3.1	1.0(0.7–1.3)

The annual rheumatology episode rate among controls is broadly similar across all panels in Fig. 1; varying little over the course of the 10-year timeframe but, as expected, being significantly higher in females than in males (p < 0.001). Far more variation is, however, evident among the cases. For DLBCL, both male and female patients had consistently higher episode rates than their corresponding controls (Fig. 1A), the difference escalating in the years leading up to diagnosis. Although not as pronounced, the pattern for MZL (Fig. 1B) is broadly similar to that of DLBCL. By contrast, the annual rates for patients with FL, CLL or MM show far less evidence of systematic variation (Fig. 1C–E); although it may be worth noting that the average episode rate for MM (Fig. 1E) is significantly raised in both males and females in the year leading up to diagnosis.

Within the DLBCL case-control group (Table 1), the OR for those with at least one rheumatology episode compared to those with none was 1.9 (95% CI 1.6–2.2); the effect being strongest among those with three or more episodes (OR 2.4, 95% CI 2.0–3.0). When data in the year before diagnosis were excluded, the association remained; the ORs being 2.3 (95% CI 1.9–2.8) and 2.3 (95% CI 1.8–2.8) respectively when data in the 6 months and 12 months before diagnosis were excluded. The pattern for MZL is broadly similar, the ORs in those with three or more episodes being 2.2 (95% CI 1.6–2.9) with no data censoring, and 2.1 (95% CI 1.5–2.8) and 2.0 (95% CI 1.5–2.7) respectively for censoring at 6 months and 12 months prior to diagnosis. However, for FL and MM, no statistically significant associations remained when the data in the 12 months before diagnosis/pseudo-diagnosis were removed. Furthermore, with most ORs being close to one, there is no evidence of any effects for CLL (Table 1).

**Fig. 2:**
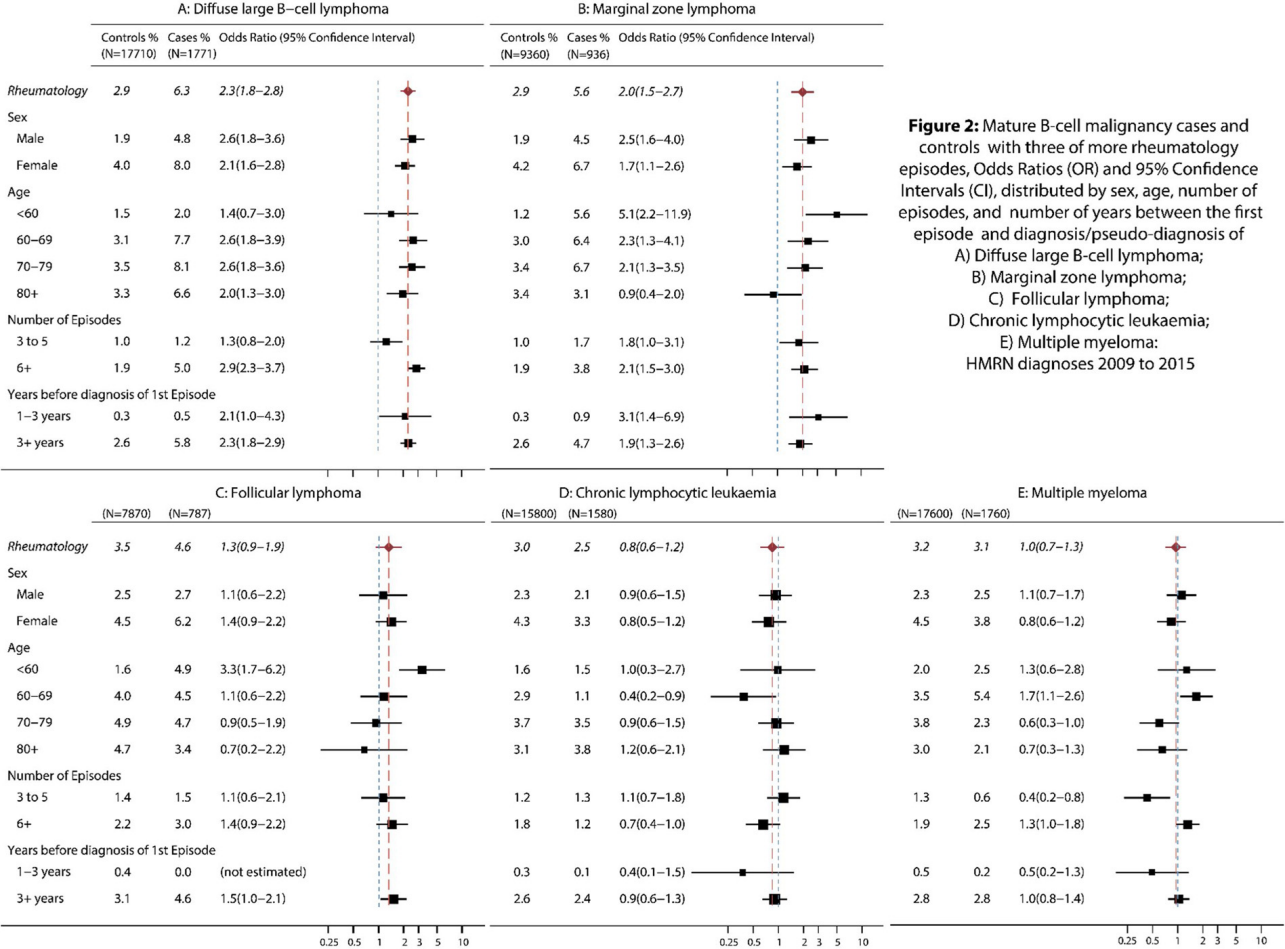
Mature B-cell malignancy cases and controls with three of more rheumatology episodes, Odds Ratios (OR) and 95% Confidence Intervals (CI), distributed by sex, age, number of episodes, and number of years between the first episode and diagnosis/pseudo-diagnosis of A) Diffuse large B-cell lymphoma; A) Marginal zone lymphoma; C) Follicular lymphoma; D) Chronic lymphocytic leukaemia; E) Multiple myeloma: HMRN diagnoses 2009 to 2015.

Comparing individuals with three or more rheumatology episodes to those with none, and censoring 12 months before cancer diagnosis, Fig. 2 presents ORs stratified by sex, age, number of episodes, and timing of the first recorded episode prior to diagnosis/pseudo-diagnosis. Although the frequency of secondary care rheumatology episodes is consistently higher for females, both among case groups and control groups, there is no evidence that sex either modifies or confounds the association with malignancy; the within subtype sex-specific ORs being similar to each other and to the overall OR (Fig. 2). Within the MZL and FL groups, there is, however, some evidence that age may be acting as an effect modifier. For MZL, the ORs range from 5.1 (95% CI 2.2–11.9) in those aged under 60 years through to 0.9 (95% CI 0.4–2) in those 80 years or more (χ^2^ = 9.40, p = 0.02). Albeit with estimates closer to unity, a similar pattern is seen for FL (χ^2^ = 9.11, p = 0.03), where the OR in those diagnosed aged<60 years stands apart from the rest, and is significantly raised (OR 3.3, 95% CI 1.7–6.2).

With a view to looking at markers of rheumatological disease severity, both the number of rheumatology episodes recorded in HES, and the time between the first recorded episode and diagnosis/pseudo-diagnosis were examined (Fig. 2). While the latter marker was not particularly discriminatory, the former provides some evidence of an effect for DLBCL; 5% of cases compared to 1.9% of controls having six or more rheumatology episodes recorded, yielding an OR of 2.9 (95% CI 2.3–3.7), which is more than twice that in the three to five episode group (OR 1.3, 95% CI 0.8–2.0). In MZL, ORs were raised in both groups, with the OR for six or more episodes being only slightly greater than that for three to five episodes (OR = 2.1, 95% CI 1.5–3.0; OR = 1.8, 95% CI 1.0–3.1 respectively).

To examine the potential impact of rheumatological disease on outcome following the diagnosis of malignancy, Table 2 compares the overall survival (OS) of cases with three or more rheumatology episodes to that of those with none. For DLBCL, the 1-year and 3-year OS of patients with three or more preceding rheumatology episodes were 59.5% and 46.6%, respectively, which was significantly poorer than that of those with no episodes (68.3% and 58.5%, respectively); the corresponding Hazard Ratios (HRs) being 1.4 (95% CI 1.0–1.9, p = 0.04) at 1-year, and 1.4 (95% CI 1.1–1.9, p = 0.01) at 3-years. However, the 111 patients with three or more episodes were, on average, significantly older than the 1597 who had none (median ages 73.0 and 70.3 years, respectively); and, when age was adjusted for, the 1-year and 3-year HRs dropped to 1.2 (95% CI 0.9–1.6, p = 0.27) and 1.2 (95% CI 0.9–1.6, p = 0.12) respectively. No other statistically significant survival differences in outcome were detected.

**Table 2:** Numbers, median ages, Overall Survival (OS) and Hazard Ratios (HR) of patients with no rheumatology episodes, and those with three or more rheumatology episodes distributed by diagnostic category (episodes in the year before diagnosis are excluded): HMRN diagnoses 2009–2015, followed-up to 05/07/2018.

	Cases, N(%)	Age^1^, median(IQR)	1-year OS, % (95%CI)^2^	HR(95%CI), unadjusted	HR(95%CI), adjusted^3^	3-year Overall Survival^1^, % (95%CI)	HR(95%CI), unadjusted	HR(95%CI), adjusted^3^
*Diffuse large B-cell lymphoma*	*1771(100.0)*	*70.6(61.1*–*79.1)*	*67.4(65.2*–*69.5)*			*57.4(55.1*–*59.7)*		
Rheumatology episodes								
None	1597(90.2)	70.3(60.7–78.9)	68.3(65.9–70.5)	1(ref)	1(ref)	58.5(56.0–60.8)	1(ref)	1(ref)
≥3	111(6.3)	73.0(66.3–79.7)	59.5(49.7–67.9)	1.4(1.0–1.9)	1.2(0.9–1.6)	46.6(37.0–55.6)	1.4(1.1–1.9)	1.2(0.9–1.6)
		Z=−3.01, p < 0.01	χ^2^ = 4.32, p = 0.04			χ^2^ = 6.80, p < 0.01		

*Marginal zone lymphoma*	*936(100.0)*	*72.4(64.4*–*79.5)*	*90.1(88.0*–*91.8)*			*74.5(71.6*–*77.2)*		
Rheumatology episodes								
None	840(89.7)	72.6(64.6–79.6)	90.1(87.9–92.0)	1(ref)	1(ref)	74.5(71.4–77.3)	1(ref)	1(ref)
≥3	52(5.6)	70.9(63.0–77.6)	90.4(78.4–95.9)	1.0(0.4–2.4)	1.3(0.5–3.2)	78.8(64.9–87.6)	0.8(0.4–1.5)	1.0(0.6–1.9)
		Z = 1.41, p = 0.16	χ^2^ = 0.01, p = 0.96			χ^2^ = 0.40, p = 0.53		

*Follicular lymphoma*	*787(100.0)*	*65.3(56.2*–*73.8)*	*94.8(93.0*–*96.1)*			*85.4(82.7*–*87.7)*		
Rheumatology episodes								
None	726(92.2)	65.6(56.2–73.8)	94.6(92.7–96.0)	1(ref)	1(ref)	85.0(82.1–87.4)	1(ref)	1(ref)
≥3	36(4.6)	63.0(56.8–75.8)	97.2(81.9–99.6)	0.5(0.1–3.7)	0.6(0.1–4.4)	91.1(75.0–97.1)	0.5(0.2–1.7)	0.6(0.2–1.9)
		Z = 0.41, p = 0.68	χ^2^ = 0.45, p = 0.50			χ^2^ = 1.20, p = 0.28		

*Chronic lymphocytic leukaemia*	*1580(100.0)*	*71.1(62.9*–*78.9)*	*90.9(89.4*–*92.2)*			*79.9(77.8*–*81.8)*		
Rheumatology episodes								
None	1490(94.3)	71.0(62.8–78.9)	90.7(89.2–92.1)	1(ref)	1(ref)	80.2(78.0–82.1)	1(ref)	1(ref)
≥3	40(2.5)	75.7(70.6–82.1)	92.5(78.5–97.5)	0.8(0.3–2.6)	0.6(0.2–1.9)	67.2(50.3–79.5)	1.8(1.0–3.1)	1.4(0.8–2.4)
		Z=−2.71, p < 0.01	χ^2^ = 0.11, p = 0.74			χ^2^ = 4.30, p = 0.04		

*Multiple myeloma*	*1760(100.0)*	*72.6(64.3*–*79.8)*	*75.1(73.0*–*77.0)*			*55.6(53.2*–*57.9)*		
Rheumatology episodes								
None	1647(93.6)	72.7(64.5–79.8)	74.9(72.8–76.9)	1(ref)	1(ref)	56.0(53.6–58.4)	1(ref)	1(ref)
≥3	54(3.1)	67.6(63.8–76.3)	75.9(62.2–85.2)	1.0(0.6–1.7)	1.1(0.6–1.9)	48.0(34.2–60.5)	1.3(0.9–1.8)	1.4(1.0–2.1)
		Z = 1.64, p = 0.10	χ^2^ = 0.01, p = 0.92			χ^2^ = 1.40, p = 0.24		

1 Wilcoxon rank-sum test was used to compare age distributions.

2 Log rank test was used to test for survival differences.

3 Adjusted for age.

Table 3 presents additional prognostic and treatment data for DLBCL; the most aggressive of the five mature B-cell malignancies examined, and the only one that is potentially curable with standardized first-line chemotherapy. In our cohort, 80.2% (1421/1771) of DLBCL patients received first-line potentially curative treatment; those who did not tending to have a poor performance status and more advanced disease. As can be seen from Table 3, DLBCL patients with ≥3 rheumatology episodes were, however, slightly less likely (p = 0.047) to be treated with curative intent (73.0%, 81/111) than those with none (81.0%, 1294/1597). Nonetheless, no significant survival differences between those with ≥3 rheumatology episodes and those with none remained once the standard prognostic factors (age, performance status, B-symptoms and cancer stage) were accounted for; either among the total patient group (HR = 1.0, 95% CI 0.8–1.3) or among the 80.0% who were treated with curative intent (HR = 0.9, 95% CI 0.6–1.3).

**Table 3:** Total number of patients with diffuse large B-cell lymphoma, number with no rheumatology episodes, and number three or more rheumatology episodes distributed by prognostic factors and treatment: HMRN diagnoses 2009–2015, followed-up to 05/07/2018.

	All patients			Treated with curative intent		
		Rheumatology episodes			Rheumatology episodes	
	N (%)	None (%)	3 or more (%)	N (%)	None (%)	3 or more (%)
Total	1771(100.0)	1597(100.0)	111(100.0)	1421(100.0)	1294(100.0)	81(100.0)

Age, median (IQR)	70.6 (61.1–79.1)	70.3 (60.7–78.9)	73.0 (66.3–79.7)	68.3 (59.5–76.6)	68.0 (58.9–76.3)	71.1 (65.8–78.3)
		Z=−3.01, p < 0.01			Z=−3.05, p < 0.01	

Performance status						
0- Good	567(32.0)	537(33.6)	19(17.1)	549(38.6)	520(40.2)	19(23.5)
1	583(32.9)	527(33.0)	36(32.4)	522(36.7)	469(36.2)	33(40.7)
2	356(20.1)	309(19.3)	33(29.7)	250(17.6)	218(16.8)	20(24.7)
3 & 4- Poor	205(11.6)	170(10.6)	19(17.1)	86(6.1)	75(5.8)	7(8.6)
		χ^2^ = 18.2, p < 0.01			χ^2^ = 9.72, p = 0.02	

B symptoms						
Absent	1002(56.6)	917(57.4)	50(45.0)	819(57.6)	754(58.3)	43(53.1)
Present	739(41.7)	652(40.8)	60(54.1)	602(42.4)	540(41.7)	38(46.9)
		χ^2^ = 7.10, p < 0.01			χ^2^ = 0.84, p = 0.36	

Cancer stage						
I & II	497(28.1)	454(28.4)	28(25.2)	469(33.0)	430(33.2)	25(30.9)
III & IV	925(52.2)	829(51.9)	59(53.2)	766(53.9)	689(53.2)	50(61.7)
Not Fully Staged	319(18.0)	286(17.9)	23(20.7)	186(13.1)	175(13.5)	6(7.4)
		χ^2^ = 0.85, p = 0.65			χ^2^ = 3.32, p = 0.19	

3-year OS (95% CI)	57.4 (55.1–59.7*)*	58.5 (56.0–60.8)	46.6 (37.0–55.6)	68.2 (65.7–70.6)	68.6 (66.0–71.1)	62.6 (51.0–72.2)
		χ^2^ = 6.77, p < 0.01			χ^2^ = 1.12, p = 0.29	

Fully adjusted HR^1^(95%CI)		1(ref)	1.0(0.8–1.3)		1(ref)	0.9(0.6–1.3)

1 Hazard ratios adjusted for age, performance status, B symptoms, and stage.

With a view to gaining further insight into the association between B-cell lymphomas and rheumatological disorders, we examined relationships with cancer site for MZL and DLBCL, and cell-of-origin for DLBCL. With respect to the former, as would perhaps be expected, the site distribution of patients with MZL who had ≥3 rheumatology episodes differed from that of those with none (p = 0.015, data not shown); the difference being partly driven by the relatively high proportion of patients in the rheumatoid group with MZL tumours in the salivary gland, the only individual site to vary significantly (10.6% *versus* 1.1% respectively, p = 0.002). However, whilst the exclusion of these tumours (most likely associated with Sjögren’s syndrome) reduced the OR for rheumatology (≥3 episodes *versus* none, excluding episodes in the 12 months prior to diagnosis/pseudo-diagnosis) the effect remained (OR = 1.8, 95% CI 1.3–1.4). The site distribution also differed for DLBCL (p = 0.007); the largest difference being the excess involvement seen for the rheumatoid group within the epitrochlear nodes of the arm, the only individual site to vary significantly (2.9% *versus* 0.1%, p = 0.026). The numbers involved were, however, small (n = 4) and their removal had no impact on the risk estimate.

With respect to cell-of-origin, 47.8% (816/1708) of DLBCL patients had sufficient immunohistochemistry (CD10, BCL6 and IRF4/MUM1) to enable their tumour to be classified as either GCB or non-GCB; 47.6%, (761/1597) of those with no rheumatology episodes, and 49.5% (55/111) of those with three or more episodes. The proportions of GCB and non-GCB were similar; GCB accounting for 55.5% (423/761) in those with no rheumatology episodes and 56.5% (31/55) in those with three or more (p = 0.91). Within the DLBCL subgroup of 505 (29.6%) that could be categorized on the basis of gene-expression profiling (GCB, ABC, or unclassified), the findings were similarly negative (data not shown).

## 4 Discussion

Incorporating data on nearly 7000 patients newly diagnosed with one of the five commonest mature B-cell malignancies and ten times as many controls, this large UK population-based record-linkage study confirmed the well-known association between rheumatological disorders and subsequent DLBCL and MZL development [5,7,15]. By contrast, highlighting the heterogeneity of this diverse cancer group, no associations were observed for FL, CLL or MM; again broadly agreeing with the few studies that have reported on this topic [7,30,31]. Interestingly, however, even though as expected women were significantly more likely than men to have rheumatological episodes, there was little indication that sex impacted on the strength of the association with B-cell malignancy. However, we did find evidence to support the suggestion that DLBCL risk was increased among those (males and females) with more severe rheumatological disease; which, in addition to being consistent with a potential aetiological role for chronic inflammation, is also consistent with the suggestion that intensive immunosuppressive therapies could have a part to play, although the evidence on this topic is conflicting [15,[32], [33], [34]]. Importantly, our findings mitigate against the view that the association between DLBCL and chronic B-cell activating inflammatory diseases is likely to be mediated by an increase in the incidence of the ABC subtype: no cell-of-origin frequency differences with rheumatology status being observed for either of the classification schemas applied to patients with available samples (816/1708). Furthermore, no systematic survival differences with rheumatology status were found for DLBCL, or indeed any of the five malignancies examined. In this context, it is relevant to note that the cell-of-origin assignment methods used in the present report are associated with large survival differences in our DLBCL patient cohort, and that this effect remains even after adjustment for a wide range of established prognostic factors [13].

Major strengths of our study include its large well-defined catchment population; the socio-demographic structure of which, at around 4 million, accounts for around 6% of the UK’s estimated total and is broadly representative of the national population as a whole in terms of age, sex, and deprivation [1,19,20]. HMRN’s patient cohort, which sits within this population, was initiated with the specific aim of producing “real-world” generalizable data to inform contemporary clinical practice and research. Importantly, clinical practice across the region adheres to national guidelines and, in contrast to most population-based registers, all patients in the study area benefit from world-class centralised diagnostics; ensuring accuracy and consistency in the diagnostic process, as well as completeness of cancer ascertainment. Sourced from the same catchment population as the patient cohort, and linked to the same nationwide administrative databases (deaths, cancer registrations, and HES), HMRN’s comparison cohort was specifically constructed to enable robust comparisons to be made between patients with haematological malignancies and individuals from the general population. Furthermore, our use of clinical specialty delivering patient care is likely to have captured the majority of individuals with active rheumatological disease since, as detailed in the methods, the diagnosis and treatment of autoimmune/inflammatory conditions in the UK requires specialist clinical input; patients are both diagnosed and managed as outpatients in secondary care [27,28]. Indeed, this is evidenced by the strong patterns seen within our data, where virtually all “exposed” individuals attending an outpatient rheumatology clinic did so on three or more occasions.

With respect to weaknesses, while the diagnostic and treatment details relating to cancer subtypes in HMRN’s patient cohort are superior to most studies, using HES data to categorize “exposure” (in this case those with and without rheumatological disorders) is clearly less robust; albeit not subject to the biases commonly associated with studies based on self-reported illness histories [10,16,17,30,35]. Nevertheless, the fact remains that we could not directly identify different rheumatological disease subtypes, dates of diagnosis, or treatments. Furthermore, although censoring 12 months before diagnosis/pseudo-diagnosis hopefully reduced the potential for detection bias resulting from reverse causality, it is possible that some weaker associations may have been obscured. The timeframe over which we could investigate was also limited by the fact that outpatient HES is only available from 2003 onwards and, given the nature of the relationship being investigated, a longer period would have been preferable. The analyses presented here have, however, demonstrated the utility of HES data for investigations of this type; and it is possible that changes in national recording procedures may facilitate more detailed analyses in the future.

## 5 Conclusions

Our findings support the hypothesis that the chronic activation and proliferation of specific B-cell populations which characterize autoimmune diseases like rheumatoid arthritis and Sjögren’s syndrome, increase the potential for the lymphomagenic events that lead to DLBCL and MZL in both males and females; but have no impact on the development of CLL, FL or MM. In addition, for DLBCL, whilst our findings provide evidence for an association with rheumatological disease severity, they offer little support for the notion that the association is driven by an increase in the ABC subtype. More importantly, perhaps, the observation that individuals with chronic inflammatory disorders are not additionally disadvantaged with respect to cancer survival, offers some reassurance to patients with these conditions, as well as to the clinicians who treat them.

## Author contributions

ER and EK drafted the manuscript. EK, DP and AS managed the data and carried out the analyses, SC provided additional statistical input, SO provided public health input, and RP provided clinical advice. All authors contributed to discussions and the final draft of the paper.

## Funding

The Haematological Malignancy Research Network’s patient cohort is funded by Bloodwise (grant number 15037), and its comparison cohort by CRUK (grant number C9474/A18362). The funders had no involvement in the conduct of the research.

## Ethics

The research has ethics approval (REC 04/01/1205/69) from Leeds West Research Ethics Committee, R&D approval from each NHS Trust and exemption from Section 251 of the Health & Social Care Act (PIAG 1-05(h)/2007).

Declarations of interest

None.
